# The complete mitochondrial genome of a non-biting midge *Polypedilum unifascium* (Tokunaga, 1938) (Diptera: Chironomidae)

**DOI:** 10.1080/23802359.2021.1945977

**Published:** 2021-07-05

**Authors:** Teng Lei, Chao Song, Xu-Dong Zhu, Bin-Ye Xu, Xin Qi

**Affiliations:** a College of Life Sciences, Taizhou University, Taizhou, China; b Forestry Administration of Cangxi Country, Guangyuan, China

**Keywords:** Chironomidae, mitochondrial genome, phylogeny, *Polypedilum unifascium*

## Abstract

The complete mitochondrial genome of *Polypedilum unifascium* (Diptera: Chironomidae) was determined by Illumina sequencing technology. The whole mitogenome is 16,452 bp in length with an A + T bias of 79.3%, and contains 13 protein-coding genes (PCGs), 22 transfer RNAs (tRNAs), and two ribosomal RNAs (rRNAs). All PCGs start with ATN codon and use TAA as the stop codon. Gene arrangement of the 13 PCGs is identical to that of other known Chironomidae mitochondrial genomes. The resultant Bayesian inference and maximum-likelihood trees based on the sequence data of 13 PCGs support its close relationship with *P. vanderplanki*.

The aquatic chironomids, commonly known as non-biting midges are often the dominant group among freshwater benthic invertebrates. The family Chironomidae consists of diverse species which are used as biomonitors of water quality (Saether 1979). The species identification and phylogenetic analysis of chironomids mostly rely on morphologic characteristics, as well as molecular makers especially mitochondrial genomes. Nevertheless, only three valid mitochondrial genomes have been deposited in public database GenBank of NCBI. In a biodiversity survey at Lishui, China, a species of Chironomidae, *Polypedilum unifascium*, was found. *P. unifascium* was first described in Japan, and continuously recorded in Korea, China, and Russian Far East (Zhang et al. 2016; Song et al. 2017). So far, mitochondrial genome of this species has not been reported.

This article reports the complete mitochondrial genome of *P. unifascium*. The larvae were collected from fresh water sediment at Lishui, Zhejiang, China (27°45′16″N, 119°11′15″E) on August 2020. The specimens are deposited at the College of Life Sciences, Taizhou University (www.tzc.edu.cn, Xin Qi, qixin0612@tzc.edu.cn) under the voucher number BSZ13. Genomic DNA was extracted from the abdomen of a larva using DNeasy Blood & Tissue Kit (Qiagen, Hilden, Germany) and subjected to conduct next-generation sequencing at Illumina NovaSeq 6000 platform. The raw reads were filtered by Trimmomatic version 0.39 (Bolger et al. 2014) and the clean reads were assembled by SPAdes version 3.14.1 (Bankevich et al. 2012). The assembly mitochondrial genome sequence was annotated with MITOS web server (Bernt et al. 2013) and tRNAscan-SE (Lowe and Chan 2016). Some annotations were corrected manually.

The complete mitochondrial genome of *P. unifascium* is 16,452 bp in length, and contains 13 protein-coding genes (PCGs), 22 transfer RNAs (tRNAs), and two ribosomal RNAs (rRNAs). The overall base composition is 40.4% A, 38.9% T, 7.9% G, and 12.8% C. All the PCGs start with ATN codon, and use TAA as the stop codon. Gene arrangement of the 13 PCGs is identical to that of other known Chironomidae mitochondrial genomes.

To reveal the phylogenetic position of *P. unifascium*, nucleotide sequences of the 13 PCGs from *P. unifascium* and 10 closely related Culicomorpha species, as well as an outgroup *Ptychoptera minuta* (Ptychopteromorpha; MT410803), were used to construct phylogenetic trees. The sequences were multiple aligned using MUSCLE (Edgar 2004) in software MEGA version X (Kumar et al. 2018), and conserved blocks were identified by Gblocks version 0.91b (Talavera and Castresana 2007). The phylogenetic relationships were reconstructed using the Bayesian inference and maximum-likelihood methods through MrBayes version 3.2.6 (Huelsenbeck and Ronquist 2001) and RAxML version VI (Stamatakis 2014). The resultant trees shared the same topologies and revealed that *P. unifascium* belonged to Chironomidae and was in close association with *P. vanderplanki* (Figure 1). This work provides molecular characterizations of *P. unifascium* and contributes to the phylogenetic analysis of Chironomidae.

**Figure 1.**
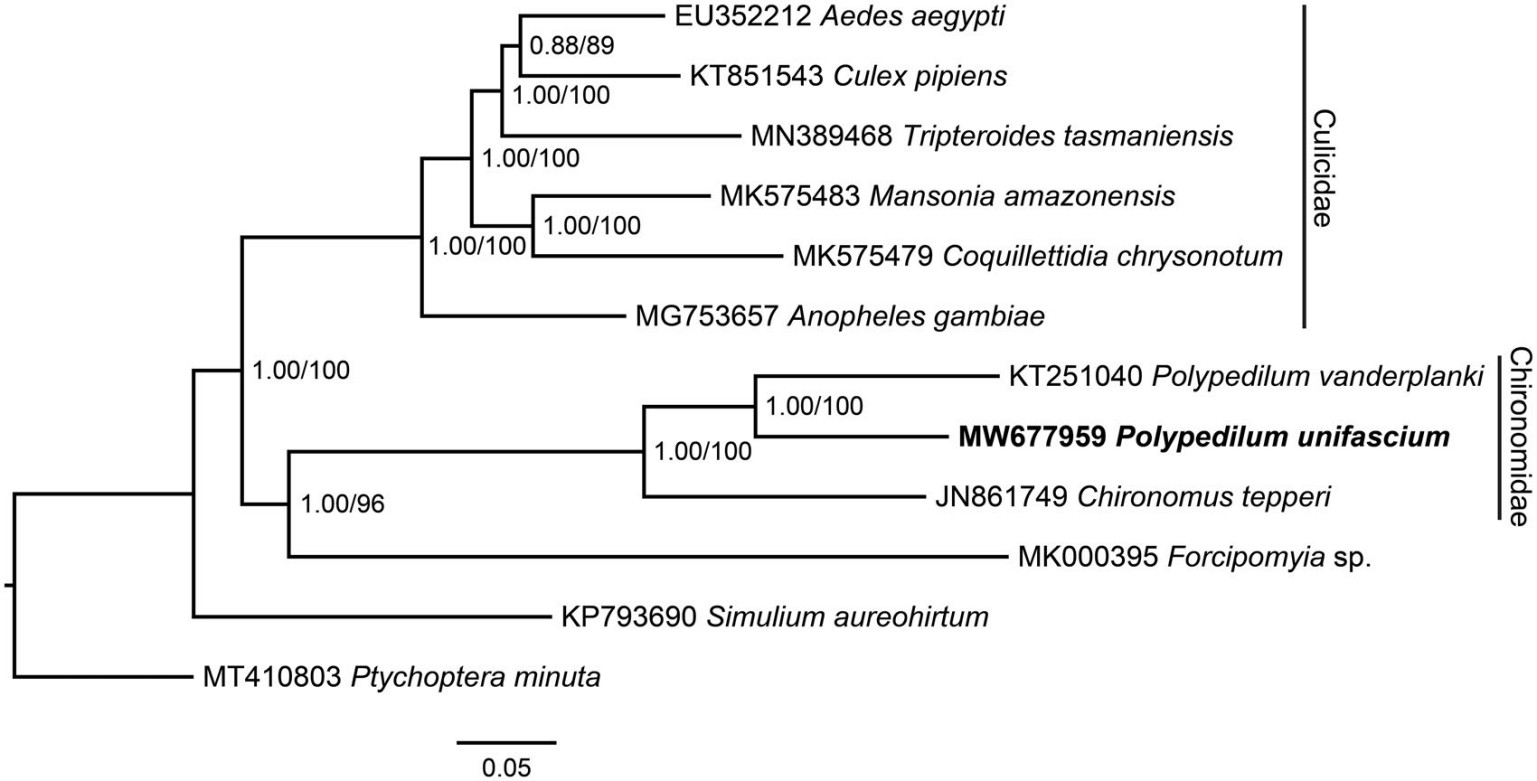
Bayesian inference and maximum-likelihood phylogenetic trees inferred from the nucleotide sequence data of mitogenomic 13 PCGs. The numbers at the nodes indicate the posterior probability values of Bayesian inference tree and the bootstrap values of maximum likelihood tree.

## Data Availability

The data support the findings of this study are openly available in GenBank of NCBI at https://www.ncbi.nlm.nih.gov. The complete mitochondrial genome of *Polypedilum unifascium* for this study has been deposited in GenBank with accession number MW677959. The associated BioProject, BioSample, and SRA numbers are PRJNA705522, SAMN18091136, and SRS8339075.

## References

[CIT0001] Bankevich A, Nurk S, Antipov D, Gurevich AA, Dvorkin M, Kulikov AS, Lesin VM, Nikolenko SI, Pham S, Prjibelski AD, et al. 2012. SPAdes: a new genome assembly algorithm and its applications to single-cell sequencing. J Comput Biol. 19(5):455–477. 22506599 10.1089/cmb.2012.0021 PMC3342519

[CIT0002] Bernt M, Donath A, Jühling F, Externbrink F, Florentz C, Fritzsch G, Pütz J, Middendorf M, Stadler PF. 2013. MITOS: improved *de novo* metazoan mitochondrial genome annotation. Mol Phylogenet Evol. 69(2):313–319. 22982435 10.1016/j.ympev.2012.08.023

[CIT0003] Bolger AM, Lohse M, Usadel B. 2014. Trimmomatic: a flexible trimmer for Illumina sequence data. Bioinformatics. 30(15):2114–2120. 24695404 10.1093/bioinformatics/btu170 PMC4103590

[CIT0004] Edgar RC. 2004. MUSCLE: multiple sequence alignment with high accuracy and high throughput. Nucleic Acids Res. 32(5):1792–1797. 15034147 10.1093/nar/gkh340 PMC390337

[CIT0005] Huelsenbeck JP, Ronquist F. 2001. MRBAYES: Bayesian inference of phylogenetic trees. Bioinformatics. 17(8):754–755. 11524383 10.1093/bioinformatics/17.8.754

[CIT0006] Kumar S, Stecher G, Li M, Knyaz C, Tamura K. 2018. MEGA X: molecular evolutionary genetics analysis across computing platforms. Mol Biol Evol. 35(6):1547–1549. 29722887 10.1093/molbev/msy096 PMC5967553

[CIT0007] Lowe TM, Chan PP. 2016. tRNAscan-SE On-line: integrating search and context for analysis of transfer RNA genes. Nucleic Acids Res. 44(W1):W54–W57. 27174935 10.1093/nar/gkw413 PMC4987944

[CIT0008] Saether OA. 1979. Chironomid communities as water quality indicators. Hol Ecol. 2(2):65–74.

[CIT0009] Song C, Liu W, Zhang R, Wang X. 2017. Two gynandromorphic species of *Polypedilum* Kieffer, 1912 (Diptera: Chironomidae), with DNA barcodes from Oriental China. Pan-Pac Entomol. 93(2):95–104.

[CIT0010] Stamatakis A. 2014. RAxML version 8: a tool for phylogenetic analysis and post-analysis of large phylogenies. Bioinformatics. 30(9):1312–1313. 24451623 10.1093/bioinformatics/btu033 PMC3998144

[CIT0011] Talavera G, Castresana J. 2007. Improvement of phylogenies after removing divergent and ambiguously aligned blocks from protein sequence alignments. Syst Biol. 56(4):564–577. 17654362 10.1080/10635150701472164

[CIT0012] Zhang RL, Song C, Qi X, Wang XH. 2016. Taxonomic review on the subgenus Tripodura Townes (Diptera: Chironomidae: Polypedilum) from China with eleven new species and a supplementary world checklist. Zootaxa. 4136(1):1–53. 27395703 10.11646/zootaxa.4136.1.1

